# Burned-out Post Pubertal Teratoma Presenting as a Liver Metastases in a 34-Year-Old Male

**DOI:** 10.30699/IJP.2020.128407.2402

**Published:** 2020-12-21

**Authors:** Fereshteh Ameli, Pooneh Panahi, Vahid Soleimani

**Affiliations:** 1 *Department of Pathology, Cancer Institute, Imam Khomeini Hospital Complex, Tehran University of Medical Sciences, Iran *

**Keywords:** Burned out, Germ cell tumor, Teratoma, Testicular tumor

## Abstract

Germ cell teratomas belong to non-seminomatous germ cell tumors and account for 95% of malignant testicular tumors. Regarding the current World Health Organization (WHO) criteria, testicular teratomas are divided into prepubertal and post-pubertal subtypes based on patients’ age. The term “burned-out testicular tumor” is a very rare condition referring to a regressed testicular tumor which presents with its metastases without any clinical finding in the testicle. Metastasis can be the presentation of post-pubertal teratoma in 22-37% of cases. In scar associated teratoma (burn-out component), the metastasis rate is 66%. We reported a rare case of post-pubertal teratoma in a 34-year-old male who presented with multiple liver masses initially. Liver biopsy revealed poorly differentiated adenocarcinoma probably of gastrointestinal (GI) tract origin. The upper and lower GI endoscopy were normal. Scrotal ultrasonography showed a hypoechoic cystic intratesticular lesion in the left testis. The patient underwent radical orchiectomy and the histopathologic examination revealed post-pubertal teratoma with burned out component. The patient underwent proper treatment and is still under follow up. As a result, in a young male patient who presented with a retroperitoneal mass or poorly differentiated carcinomas of an unknown primary site, using light microscopy and immunohistochemical profiling alone may be inadequate. Therefore, scrotal screening and physical examination of the scrotum and bilateral testis should be considered to exclude possibility of a metastatic progression from a testicular germ cell neoplasia.

## Introduction

Germ cell tumors (GCTs) account for 95% of the malignant testicular tumors. Seminomas tumors consist 40% to 50% of GCTs (1). Non-seminomatous GCTs include embryonal cell carcinoma (20-25%), teratoma (5-10%), choriocarcinoma (1-3%), and mixed tumors (20-40%), which is a mixture of virtually all histological types (2).

Testicular teratomas are divided into prepubertal and post-pubertal subtypes based on the current World Health Organization (WHO) criteria. This categorization generally corresponds to the patients’ age in relation to puberty; however, age categorization of prepubertal-type teratoma results in combining tumors with different pathogenesis and clinical behaviors (1). Post-pubertal teratoma is more frequent than prepubertal teratoma (3).

It is well recognized that a small subset of testicular germ cell tumors, including teratomas, undergo partial or complete regression (<5%), which results in a well-defined nodular scar or fibrosis in testis. These patients may present with metastases and nonspecific findings on testicular ultrasound. Histologically, regression is characterized by a fibrotic scar with variable lymphoplasmacytic inflammation, ischemic “ghosts” of hyalinized tubules, increased number of small blood vessels, hemosiderin-laden macrophages and coarse intratubular calcifications (3).

The phenomenon of a primary testicular germ cell tumor (TGCT) outgrowing its blood supply and undergoing auto-infarction has been described as a ‘burned out’ TGCT. Despite the spontaneous regression of the primary testicular tumor, approximately 50% of ‘burned out’ primary testicular tumors continue to harbor malignant cells and distant metastatic disease and can progress. The metastases of testicular germ cell tumors mainly develop through lymphatic route. Only pure choriocarcinoma is spread through haematogenic pathway (4).

It may be difficult to determine the source of metastasis when the testicular tumor is totally regressed (5). Identifying testis as the origin of metastasis is crucial as there is a high chance for testicular relapse if orchiectomy is not performed (1).

Here we discuss a case of post-pubertal teratoma with ‘burned out’ component presenting as multiple liver masses. The importance of this case is related to the rare presentation of this phenomenon and the importance of carrying out a systematic testicular ultrasound examination in case of metastasis in a young man.

## Case Presentation

**Fig. 1:**
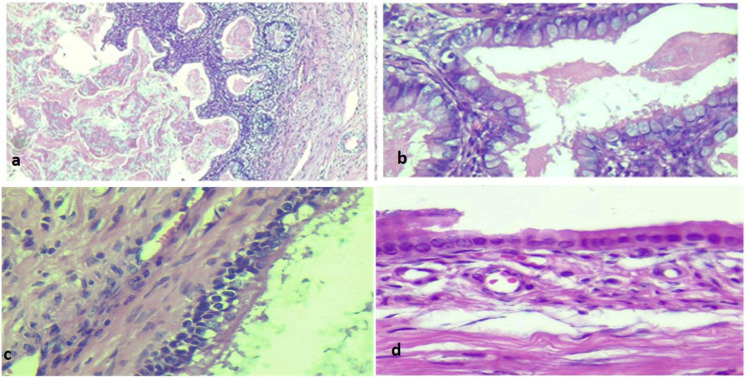
Hematoxylin and eosin staining showing cystic structures lined by intestinal epithelium (a: ×100, b: ×400), respiratory epithelium (c: ×400) and focused apocrine metaplasia (d: ×400)

**Fig. 2:**
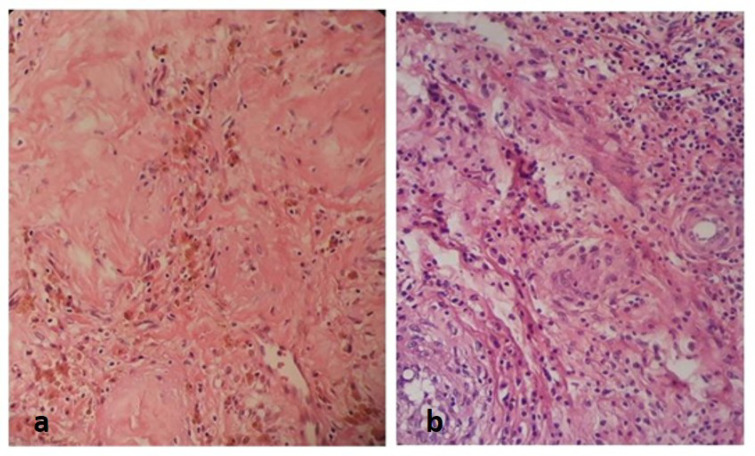
Hematoxylin and eosin staining showing a focus of fibrotic scar with mild infiltration of lymphoplasma cells, aggregates of hemosiderin laden macrophages and increased number of small vessels consistent with burned out components at the periphery of the cystic lesion

**Fig. 3:**
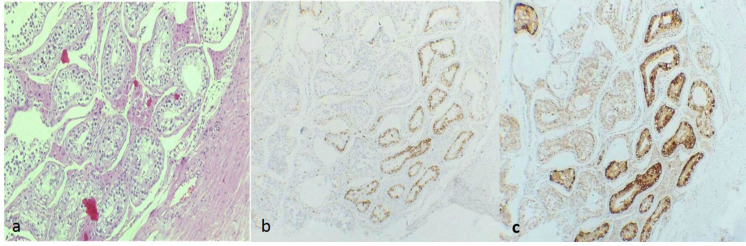
a. Hematoxylin and eosin staining showing some foci of intra-tubular germ cell neoplasia in the surrounding testicular tissue (×100). Immunohistochemistry for PLAP (b) and CD117 (c), highlighting an intra-tubular germ cell neoplasia (×100).

A 34-year-old man presented with left upper quadrant pain. An abdominal computed tomography (CT) scan and sonography showed multiple liver masses. He underwent liver core needle biopsy in another center and based on immunohistochemistry study (positivity for CK20 and CDX2 with negativity of CK7) the diagnosis was a poorly differentiated adenocarcinoma probably originated from GI tract. Therefore, he underwent multidisciplinary investigations to elucidate tumor origin, including upper and lower GI endoscopy and abdominal and lung CT scan. All of the assessments as well as laboratory testing for tumor markers, including testicular tumor markers, were normal. There was no abnormality in the physical examination of the scrotum. However, scrotal ultrasonography showed a hypoechoic cystic intratesticular lesion in the left testis. He underwent a radical left orchiectomy. On gross examination, the testicle measured 5×3.5×3 cm. Tunica vaginalis and albuginea and spermatic cord showed no gross abnormalities. Cut surface revealed a well-defined multicystic lesion measuring 2×1×0.8 cm, confined to the testis. Microscopic examination exhibited multicystic spaces lined by gastrointestinal and respiratory type epithelium with foci of apocrine metaplasia (Figure 1, a-d). Areas of fibrotic scar with mild lymphoplasmacytic infiltrate and increased number of small blood vessels were also identified at the periphery of the lesion (Figure 2, a-b). Intratubular germ cell neoplasia was found in surrounding testicular tissue, which were confirmed by immunohistochemistry for PLAP and CD117 (Figure 3, a-c). These findings were consistent with post-pubertal teratoma with burned out component. Thus, a diagnosis of spontaneous regression of a germ cell tumor was made. Later, he received three cycles of chemotherapy and still is under follow up. 

## Discussion

Although testis cancer is rare (1% of all cancers in men), it is the most common cancer in young men. Majority (90 % to 95%) of all primary testicular tumors develop from germ cells (1). Germ cell tumors include seminomatous tumors, non-seminomatous tumors (embryonic carcinoma, tumor of the yolk sac, teratoma, choriocarcinoma) and mixed forms (4).

Based on the current WHO criteria, testicular teratomas are divided into prepubertal and pos-tpubertal subtypes (1). Post-pubertal type teratoma is a malignant germ cell tumor composed of several types of tissues, including one or more of the germinal layers (1). It may be exclusively composed of a well-differentiated, mature tissue in spite of its malignant behavior. The most common tissues observed in testicular post-pubertal teratoma include neural, cartilaginous, and various types of epithelial tissues (6).

Majority (approximately 90%) of post-pubertal testicular teratoma are associated with germ cell neoplasia in situ (GCNIS) in contrast to prepubertal type which shows no association with GCNIS (6). Furthermore, no dysgenetic parenchymal changes, scarring or chromosomal 12p amplification is seen in prepubertal type (1).

The term “burned-out testicular tumor” refers to a regressed testicular tumor which presents along with metastases (2). Prym first described this phenomenon in 1927 (4). Burned-out testicular tumors may present by metastases to the retroperitoneum, mediastinum, lymph nodes, lungs and liver (1). 

Clinical manifestations generally depend on the metastatic disease. Only a few non-metastatic cases were diagnosed with signs and local symptoms, including pain in the scrotal sack, testicular shrinking and infertility (7).

The two main hypothesized mechanisms for this regression include cytotoxic T lymphocyte mediated immunological response and ischemic response due to reduced blood supply. Cytotoxic T lymphocyte mediated immunological response recognizes tumor antigens and destroys malignant neoplastic cells with subsequent fibrosis replacement. On the other hand, ischemic response occurs in the tumor due to blood supply deficit, which might be due to one or a combination of tumor cell high metabolic rate or intermittent testicular torsion (7).

Existence of testicular tumors cannot be confirmed or excluded solely through palpation. Therefore, supplemental studies with ultrasound are mandatory (7). Ultrasound scan reveals a hyperechogenic, sometimes calcified area, corresponding to the tumoral scar, a hypo or hyperechogenic area that may be observed in contact with the lesion, corresponding to the residual tumor (4).

The sensitivity of the testicular ultrasound for diagnosing GCT is nearly 100%, therefore, all young patients with metastasis, including retroperitoneal, mediastinal or liver massed, should undergo testicular ultrasonography. The characteristics of testicular tumor regression are not specific and may include hypo or hyperechogenic area, sometimes with calcification and fibrosis corresponding to tumoral scar (7).

Tumor marker assessments are necessary in the diagnostic approach of testicular tumors, staging, treatment and follow up, depending on the histological lineage and the response to treatment (7).

The diagnosis is anatomopathological. The microscopic findings in our study were in line with the WHO diagnostic criteria for testicular tumor regression, including inflammatory lymphoplasmacytic infiltrate (90% of cases), tubular hyalinization (approximately 70% of cases), increased vascularity (50% of cases), hemosiderin (44% of cases) and thick intratubular calcifications. The non-tumoral areas of our case also showed features of atrophy and sclerosis of the seminiferous tubules (100% of cases), germinal cell malignancy in situ (approximately 50% of cases) but no hyperplasia in Leydig cells (45% of cases) or intra-tubular microliths (30% of cases) is seen (4).

In the approach of this young male patient with metastasis, the histomorpholigical results of the lesions, as well as the clinical findings, laboratory and imaging studies, allowed us to formulate the primary testicular origin of tumor as burn out post-pubertal teratoma.

## Conclusion

As a result, in a young male patient who presented with a retroperitoneal mass or metastasis, a possibility of a germ cell neoplasia should be considered and testicular evaluation including physical examination and ultrasound of the scrotum and bilateral testis in the work-up is mandatory.

## References

[1] Moch H, Cubilla AL, Humphrey PA, Reuter VE, Ulbright TM (2016). The 2016 WHO classification of tumours of the urinary system and male genital organs-part A: renal, penile, and testicular tumours. Eur Urol..

[2] El-Sharkawy MS, Al-Jibali AS (2017). Burned-out metastatic testicular tumor: Choriocarcinoma. Int J Health Sci (Qassim)..

[3] Balzer BL, Ulbright TM (2006). Spontaneous regression of testicular germ cell tumors: an analysis of 42 cases. Int J Surg Pathol..

[4] Peroux E, Thome A, Geffroy Y, Guema BN, Arnaud FX, Teriitehau CA (2012). Burned-out tumour: a case report. Diagn Interv Imaging..

[5] Johnson K, Brunet B (2016). Brain metastases as presenting feature in'burned Out'testicular germ cell tumor. Cureus..

[6] Bahrami A, Ro JY, Ayala AG (2007). An overview of testicular germ cell tumors. Arch Pathol Lab Med..

[7] Astigueta JC, Abad-Licham MA, Agreda FM, Leiva BA, De la Cruz JL (2018). Spontaneous testicular tumor regression: case report and historical review. Ecancermedicalscience..

